# Molecular analysis of dengue 3 viruses detected from dengue outbreak areas in southern Vietnam in 2010

**DOI:** 10.1186/1753-6561-5-S1-P50

**Published:** 2011-01-10

**Authors:** Vu Thi Que Huong, Vu Dinh Luan, Huynh Phuong Thao, Vu Thien Thu Ngu

**Affiliations:** 1 Pasteur Institute of Ho Chi Minh City, Vietnam

## 

There are four dengue virus serotypes, based on molecular analysis. Each serotype is divided to several different genotypes, such as there are 5 genotypes in dengue virus serotype 3 (DENV-3), in which genotype II was confirmed to be the main etiology cause the big dengue fever/dengue hemorrhagic fever outbreak in Vietnam 1998.

Molecular epidemiological study was carried out on DENV-3, which causing dengue fever/dengue hemorrhagic fever in some epidemic areas with high morbidity and mortality from southern Vietnam in 2010. In this study, viral RNA of DENV-3 were extracted directly from dengue fever/dengue hemorrhagic fever patient sera, typing by RT-PCR (reverse transcriptase-polymerase chain reaction) with Lanciotti specific primers, sequencing the entire envelope (E) gene and comparative analysis with DENV-3 strain isolated from the big outbreak of dengue hemorrhagic fever in whole country in 1998. The phylogenetic analysis of DENV-3 from Vietnam as well as previously published strains, the results showed that: Vietnamese DENV-3 belong to genotype II among 5 genotypes classified for worldwide DENV-3 and have been closely related to Thailand isolates. Further researchs need to be carried out in order to confirm the evidence for local genetic evolution and conserved functional structure of E protein of DENV-3.

